# The Enigmatic Etiology of Oculo-Auriculo-Vertebral Spectrum (OAVS): An Exploratory Gene Variant Interaction Approach in Candidate Genes

**DOI:** 10.3390/life12111723

**Published:** 2022-10-28

**Authors:** Bernardette Estandia-Ortega, Miriam Erandi Reyna-Fabián, José Antonio Velázquez-Aragón, Ariadna González-del Angel, Liliana Fernández-Hernández, Miguel Angel Alcántara-Ortigoza

**Affiliations:** 1 Molecular Biology Laboratory, Medical Research Sudirectorate, National Institute of Pediatrics, Mexico City 04530, Mexico; 2 Biology Sciences, Posgrade Unit, National Autonomous University of Mexico, Mexico City 04510, Mexico; 3 Experimental Oncology Laboratory, Experimental Medicine Subdirectorate, National Institute of Pediatrics, Mexico City 04530, Mexico

**Keywords:** gene–gene interactions, microtia, Mexican population, Multifactor Dimensionality Reduction (MDR), next-generation sequencing, OAVS

## Abstract

The clinical diagnosis of oculo-auriculo-vertebral spectrum (OAVS) is established when microtia is present in association with hemifacial hypoplasia (HH) and/or ocular, vertebral, and/or renal malformations. Genetic and non-genetic factors have been associated with microtia/OAVS. Although the etiology remains unknown in most patients, some cases may have an autosomal dominant, autosomal recessive, or multifactorial inheritance. Among the possible genetic factors, gene–gene interactions may play important roles in the etiology of complex diseases, but the literature lacks related reports in OAVS patients. Therefore, we performed a gene–variant interaction analysis within five microtia/OAVS candidate genes (*HOXA2*, *TCOF1*, *SALL1*, *EYA1* and *TBX1*) in 49 unrelated OAVS Mexican patients (25 familial and 24 sporadic cases). A statistically significant intergenic interaction (*p*-value < 0.001) was identified between variants p.(Pro1099Arg) *TCOF1* (rs1136103) and p.(Leu858=) *SALL1* (rs1965024). This intergenic interaction may suggest that the products of these genes could participate in pathways related to craniofacial alterations, such as the retinoic acid (RA) pathway. The absence of clearly pathogenic variants in any of the analyzed genes does not support a monogenic etiology for microtia/OAVS involving these genes in our patients. Our findings could suggest that in addition to high-throughput genomic approaches, future gene–gene interaction analyses could contribute to improving our understanding of the etiology of microtia/OAVS.

## 1. Introduction

Microtia (HP:0008551) is a congenital anomaly of heterogeneous etiology that arises from alterations in structures derived from the first and second pharyngeal arches during the embryonic period [1,2,3]. When microtia is associated with hemifacial hypoplasia (HH, HP:0011332) and ocular, vertebral, cardiac, and/or renal malformations, the diagnosis of oculo-auriculo-vertebral spectrum (OAVS, MIM #164210) is suggested. However, there is no agreement on the minimum diagnostic criteria for OAVS [4,5,6,7,8].

Microtia is a minimal expression of the OAVS clinical spectrum, since both entities have variable phenotypic expression, asymmetric involvement of facial structures, right-side predominance, and male predilection. Moreover, familial occurrence with incomplete penetrance may be seen for microtia and/or related anomalies, such as preauricular tags and pits [9,10,11].

Genetic factors and non-genetic factors have been associated with the etiology of microtia/OAVS. Prenatal exposure to alcohol, retinoids, or maternal diabetes are considered environmental causal factors of this entity [5,12,13]. The genetic contribution to the development of microtia/OAVS is supported by diverse lines of evidence, such as: (a) the identification of families with variable expression and incomplete penetrance segregating as an autosomal dominant (AD), autosomal recessive (AR), or multifactorial trait [10]; (b) the greater concordance between monozygotic versus dizygotic twins (38.5% vs. 4.5%, respectively) [14]; (c) the differences in its prevalence across ethnicities [1], such as in Hispanic (1.12/10,000), U.S.-born Hispanic (0.83/10,000), Asian (0.54/10,000) [15], Pacific Island native (4.61/10,000), and Philippine (4.77/10,000) [16] populations; (d) the finding that murine models develop microtia due to pathogenic variants (PV) in genes orthologous to those identified as mutated in some microtia patients (i.e., *Hoxa2*, *Tcof1*, *Eya1*, and *Tbx1*) [4,17,18,19,20,21]; and (e) the observation of microtia within the clinical spectrum of more than 50 chromosomal and monogenic syndromes [4,5,22]. Despite these findings, however, the etiology underlying microtia/OAVS in most patients remains unknown.

Recently, several chromosomal abnormalities associated with microtia/OAVS have been described [23]. Genetic studies using next-generation sequencing (NGS) have been performed in patients with this entity [24,25,26,27], but only in a few cases candidate genes have been identified. These include *MYT1* (MIM*600379) [23,24,25], *AMIGO2* (MIM*615690) [26], *ZYG11B* (MIM*618673) [27], *ZIC3* (MIM*300265) [28], *VWA1* (MIM*611901) [29], *SF3B2* (MIM*605591) [30], and *EYA3* (MIM*601655) [31]. Variants in these genes were identified at a low frequency, supporting the notion that this clinical spectrum has high genetic heterogeneity [32]. Interestingly, the products encoded by *MYT1* and *ZYG11B* are involved in the retinoic acid (RA) signaling pathway [23,27], and prenatal exposure to RA was reported to have a teratogenic effect with a clinical presentation similar to that of microtia/OAVS [13].

Gene–gene interactions have been shown to play important roles in the etiologies of complex diseases [33]. However, the literature lacks any report addressing potential gene interactions in patients with microtia/OAVS [34,35]. Given that Mexican and Hispanic populations show the highest prevalence of microtia worldwide [15], we performed an epistasis analysis [36] in a group of Mexican patients by the Multifactorial Dimensionality Reduction (MDR) method in five microtia/OAVS candidate genes. We assessed *HOXA2* (MIM*604685, 7p15.2)*, TCOF1* (MIM*606847, 5q32), *SALL1* (MIM*602218, 16q12.1), *EYA1* (MIM*601653, 8q13.3), and *TBX1* (MIM*602054, 22q11.21), whose selection was supported by various lines of evidence (see Table 1).

## 2. Materials and Methods

### 2.1. Study Population

Forty-nine unrelated Mexican patients (31 males, 18 females; ages 0 to 18 years old) who were evaluated between 2015 and 2019 at the National Institute of Pediatrics (Mexico) and given a clinical diagnosis of microtia/OAVS (microtia or anotia with or without HH, structural alterations in the spine and/or kidneys) were included. Patients who met the clinical criteria for microtia/OAVS but had congenital malformations distinct from those involving the spine and/or kidneys and/or an alteration in somatic growth (low or high height) or intellectual disability were excluded. Those with reported prenatal exposure to specific teratogens associated with microtia/OAVS (e.g., alcohol, retinoids, or maternal diabetes) were also excluded. Each patient underwent a systematic physical examination by clinical geneticists and a detailed family history was obtained (25 familial cases and 24 sporadic cases). Imaging studies (computed tomography of the inner ear, orthopantomography, complete spinal radiography, and renal ultrasound) were performed on the patients and their parents. Detailed clinical information related to all included patients was previously published [41]. This research protocol was approved and registered by the Ethics, Research and Biosafety Committees of the National Institute of Pediatrics (Mexico City, Mexico, registry number 004/2017).

### 2.2. NGS of the Five Microtia/OAVS Candidate Genes

Genomic DNA isolated from peripheral blood or buccal cell samples of all patients was analyzed with a targeted five-gene NGS panel consisting of *HOXA2* (NM_006735.3), *TCOF1* (NM_001135243.1), *SALL1* (NM_002968.2), *EYA1* (NM_000503.5), and *TBX1* (NM_080647.1). The studied sequences covered the promoter region, all coding exons, and the intron–exon boundaries (200 bp). NGS libraries were prepared with an IDT xGen lockdown probe customized panel kit according to the manufacturer’s protocol. Libraries were sequenced on an Illumina MiSeq 2 × 150 platform (San Diego, CA, USA) through the Admera Health Company (South Plainfield, NJ, USA). Our in-house bioinformatics analysis pipeline included a quality evaluation and trimming of low-quality raw reads, alignment against the GRCh38 human reference sequence, and calling of single nucleotide variants (SNVs) and small insertion–deletion variants. The GATK Toolkit (version 4.2.6.1) [42] and the Alamut Batch (version 1.4), Focus (version 1.0), and Visual (version 2.7.2, Interactive Biosoftware, Rouen, France) software packages were used for variant annotation and filtering. All clinically relevant variants were confirmed by Sanger automated sequencing in the index cases and their available relatives. The identified gene variants were classified according to the criteria of the American College of Medical Genetics and Genomics and the Association for Molecular Pathology (ACMG/AMP) [43].

### 2.3. Statistical and Gene Interaction Analyses

The allele frequencies of the variants identified in the five studied genes were obtained using the allelic counting method. We first determined if the variant was in Hardy–Weinberg equilibrium using the two-tailed Fisher’s exact test, which assessed whether the distribution of genotypes was as expected in our population. When information was available, an association analysis was performed to compare the genotype frequencies in our study population with those reported in Mexican individuals from Los Angeles (1000 Genomes Project, phase 3; https://www.ncbi.nlm.nih.gov/genome/gdv/, accessed on 17 May 2022) using Armitage’s trend test as applied by the deFinetti online software (https://ihg.helmholtz-muenchen.de/cgi-bin/hw/hwa1.pl, accessed on 17 May 2022).

To identify possible intergenic and/or intragenic interactions between the variants in the analyzed loci, we used the Multifactor Dimensionality Reduction (MDR) ver. 3.0.2 software (Vanderbilt University Medical School, Nashville, TN, USA) [36].

Additionally, the STRING software (http://string-db.org, accessed on 20 September 2022) was used to look for interacting partners of known craniofacial and RA-associated proteins, including those encoded by the five studied genes. The selected candidate interacting protein partners were: AMIGO2, BAPX1, CHD7, CYP26B1, EYA1, EYA3, FGF3, FGF10, FGFR2, FRAS1, FREM2, GDF3, GRIP1, GSC, HDAC1, HDAC2, HOXA2, HOXA13, HOXD13, MLL2, MYT1, PHF5A, PITX2, PLCB4, RARB, SALL1, SF3B2, SIN3B, SIX1, SIX2, TBX1, TCOF1, TFAP2A, VWA1, ZIC3, and ZYG11B.

## 3. Results

The median read depth for the five-gene panel was 639X (range 86X–1940X), and the coverage was 99.9%. Thirty-nine gene coding variants were identified in *TCOF1*, *SALL1*, *EYA1*, and *TBX1*; of them, 17 were predicted to alter the amino acid sequence (non-synonymous). These included 15 missense mutations, 1 in-frame microdeletion, and 1 in-frame microduplication that were classified as benign (B, n = 12) and likely benign (LB, n = 5) according to ACMG/AMP criteria (see Table 2). Furthermore, 22 synonymous or non-coding variants were identified in our patients (see Table 3). No variant was identified in the coding region of the *HOXA2* gene.

### 3.1. Association Analysis

When we compared the allelic frequencies (AFs) of benign (B) or likely benign (LB) variants observed in our population with those reported in Mexican individuals from Los Angeles (1000 Genomes Project), a statistically significant difference (*p* < 0.05) was observed only for the p.(Ser159del) *SALL1* variant, which was found specifically in microtia/OAVS patients (n = 5, 3 familial and 2 sporadic cases). Since the missense p.(Val1275Ile) *SALL1* variant was present in a homozygous state in all cases and all individuals of the reference group, we did not perform an association analysis for this variant (see Table 2). Comparing the AFs of synonymous variants among our patients with those of the reference group, a statistically significant difference (*p* < 0.05) was observed only for the p.(Gly587=) *TCOF1* variant (see Table 3).

### 3.2. MDR Interaction Analysis

We analyzed possible interactions among the 17 genotypes of the variants found in *TCOF1*, *SALL1*, *EYA1*, and *TBX1* for our 49 microtia/OAVS cases and those present in the 63 individuals belonging to the reference group of a Mexican population from Los Angeles (1000 Genomes Project). Five synonymous variants (*TCOF1*: p.(Thr210=); *SALL1*: p.(Pro558=) and p.(Val190=); *EYA1*: p.(Ile195=); and *TBX1*: p.(Pro45=)) could not be subjected to MDR interaction analysis due to the lack of information in the reference group.

**Figure 1:**
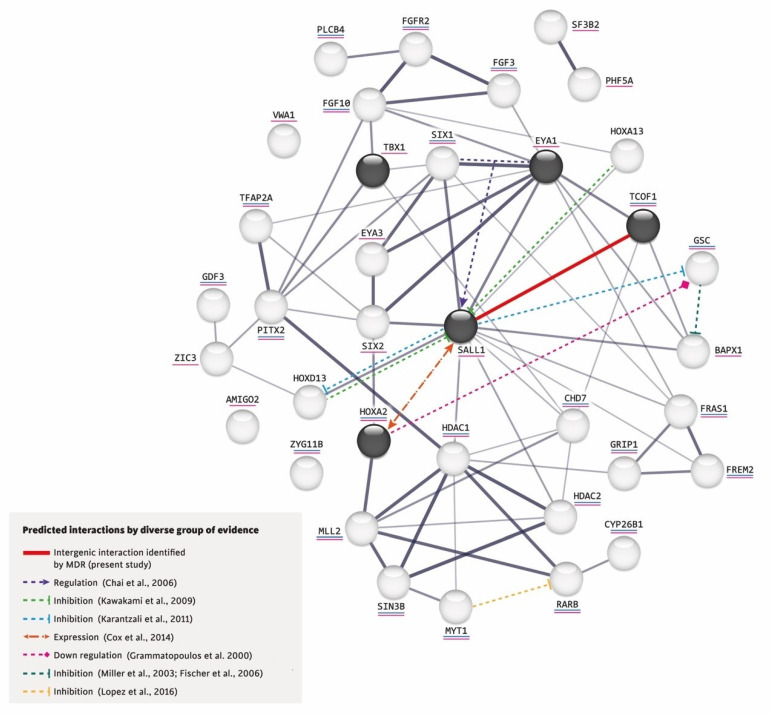
Interactions predicted by diverse lines of evidence [23,40,44,47,48,49,50,51].

One significant gene–gene interaction related to the presentation of microtia/OAVS was identified: that between the non-synonymous p.(Pro1099Arg) *TCOF1* variant and the synonymous p.(Leu858=) *SALL1* variant (Figure 1). In this MDR interaction analysis, the balance accurate cross-validation testing result was 0.8175 and the cross-validation consistency value was 10/10. In a permutation analysis performed with 10,000 repetitions, the obtained *p*-value was statistically significant (*p* < 0.001).

Furthermore, using the network created by STRING, we added the intergenic interaction that we identified in this study between the variants p.(Pro1099Arg) *TCOF1* (rs1136103) and p.(Leu858=) *SALL1* (rs1965024) and incorporated in this network the following based on reported evidence for other gene–gene interactions: (a) The co-expression of *SIX1* and *Eya1* synergistically regulates the expression of *SALL1* during kidney development [44] and also play keys roles in ear determination [45,46]. (b) In the mouse limb bud, *Hoxa13* and *Hoxd13* inhibit the expression of *Sall1* [47]. In mouse embryonic stem cells, *Sall1* appears to inhibit various *Hox* genes, including *Hoxd13* and *Gsc* [48]. (c) Although it is not known whether *Sall1* has a regulatory relationship with *Hoxa2* in the branchial arches, the former is expressed early in head mesenchyme and then becomes restricted around the first branchial cleft in close proximity of *Hoxa2*, which encodes an important transcription factor in the external ear morphogenesis of mice [40]. (d) *Hoxa2* coordinates the downregulation of *Gsc*, which acts as a transcriptional repressor in wild-type cartilage during mouse embryogenesis [49]. (e) In zebrafish, *gsc* downregulates the expression of *bapx*1 in the second pharyngeal arch [50,51]. (f) Both increased and decreased RA signaling could induce craniofacial abnormalities, such as those found in OAVS [13]. (g) *MYT1* overexpression reportedly induces the downregulation of RA receptor β (RARB), whereas mutated *MYT1* does not [23] (see Figure 1).

This interaction network was built by the STRING V.11.0 software (https://string-db.org/, accessed on 20 September 2022) and includes the proteins encoded by the five genes studied herein (in dark circles) plus those related to RA (underlined in blue) and craniofacial disorders (underlined in pink). The thickness of a gray line indicates the strength of the data compatibility based on the STRING evidence. The solid red line indicates the intergenic interaction that we herein identified between p.(Pro1099Arg) *TCOF1* (rs1136103) and p.(Leu858=) *SALL1* (rs1965024) variants, and the colored dotted lines represent the interactions documented in the literature.

## 4. Discussion

Since microtia/OAVS shows a heterogeneous etiology, incomplete penetrance, and variable expressivity, its clinical and molecular diagnoses have proven challenging. In the literature, the inclusion criteria for patients with this spectrum are diverse and not always well described or defined, which could be considered a limitation when interpreting and discussing results. We consider that the clinical inclusion criteria utilized herein allowed us to study a more homogeneous population and thereby avoid biases.

Some groups have previously analyzed genetic factors related to microtia/OAVS [22,23,24,26,27,28,52,53,54,55,56], but no previous gene–gene interaction analysis has been performed in these patients. Given the genomic differences found across populations and the high prevalence of this disorder in Latin-American populations worldwide, it is important to obtain a more precise knowledge in patients with microtia/OAVS of this ethnic origin. Gaining a better understanding of the genetic etiology of this malformation could enable clinicians to provide more accurate genetic counseling to families [17,18,19,20,21,37].

### 4.1. Association Analysis

When we performed the association study between the variants identified in our patients and the reference group, we identified that the in-frame p.(Ser159del) microdeletion of *SALL1* appears to be a risk factor for microtia, as evidenced by the statistically significant difference in the AF between our patients and the reference group and the presence of this variant in only our microtia/OAVS cases. Similar associations have been identified between variants of certain genes and the risk of developing malformations, such as congenital heart defects, biliary atresia, pyloric stenosis, hypospadias, and microtia [53,54,57].

The genome-wide association study (GWAS) approach has been successful in identifying new susceptibility loci for common structural congenital defects, such as oral clefts, congenital heart defects, biliary atresia, pyloric stenosis, hypospadias, craniosynostosis, and clubfoot [57]. However, congenital ear abnormalities, including anotia/microtia, have not previously been addressed by GWAS. The sole exception to this was a genome-wide linkage analysis performed on two families with OAVS [58]. In one family, the authors identified a highly suggestive linkage to a region harboring the *GSC* (Goosecoid homeobox) gene, which was considered to be a good candidate gene for this entity. However, coding-region changes and gross rearrangements were excluded in these two OAVS familial cases and in 120 additional sporadic cases [58].

Synonymous variants may influence the development of various human diseases, including birth defects [59,60]. A statistically significant difference was identified for the p.(Gly587=) *TCOF1* variant. The AF for this variant was greater in the reference group, suggesting that it confers protection against or a decreased risk for microtia/OAVS in our Mexican population. There is no single mechanism by which a synonymous change could exert a biological effect. Accumulating evidence shows that biological systems take advantage of the degeneracy of the genetic code to control gene expression, protein folding efficiency, and coordinated expression across several gene families. The most obvious and well-characterized mechanism by which synonymous changes can exert a deleterious biological effect is by perturbing pre-mRNA splicing [59]. However, a synonymous variant could also be in linkage disequilibrium with deleterious functional variants located nearby. To our knowledge, the p.(Gly587=) of *TCOF1* could be the first described synonymous variant associated with the microtia/OAVS trait.

### 4.2. MDR Interaction Analysis

In a very recent review of genetic and non-genetic factors involved in the development of microtia/OAVS, there was no mention of data related to gene–gene interactions [32]. As the interaction analysis for benign and/or synonymous variants could suggest a genetic protection or susceptibility factor for complex traits [34], such as ear malformations, we decided to apply an MDR analysis, which is a nonparametric model-free method for identifying epistasis using the identified variants [61]. This strategy identified a single statistically significant intergenic interaction between the non-synonymous p.(Pro1099Arg) *TCOF1* variant and the synonymous p.(Leu858=) *SALL1* variant (Figure 1). At the statistical level, combining the *TCOF1* and *SALL1* genotypes allowed us to discriminate between cases and controls, indicating that there is an interaction between these two genes. Although this interaction has not previously been reported, the TCOF1 and SALL1 proteins are related to craniofacial disorders [23] (Figure 1). The available evidence generally supports the involvement of the analyzed genes/proteins and various other genes/proteins in the development of microtia/OAVS. Notably, *SALL1* interacts with most of these genes/proteins and thus appears to play a central role. In addition, it was the gene in which the most non-synonymous variants were identified in our group of patients (Figure 1), suggesting that *SALL1* warrants future study in this regard.

Moving forward, the inclusion of a larger number of microtia/OAVS-related genes or the use of whole-exome sequencing should identify new variants that can be considered in future gene–gene interaction studies [62]. Epigenetic inheritance also has been suggested as a possible pathogenic mechanism [5]. For example, a histone acetylation-dependent imbalance in the allelic expression of the key craniofacial development gene, *BAPX1* (also called *NKX3-2*, MIM*602183), was observed in five patients with OAVS [51]. Thus, the contribution of epigenetic mechanisms to the etiology of microtia/OAVS deserves attention in future genetic studies.

The published evidence and our present findings collectively support the complexity of ear embryogenesis and microtia/OAVS development, which involves the temporal and spatial expression of different proteins and signaling by multiple pathways. We did not identify any pathogenic variant in the five studied genes, but we found a gene–gene interaction between *TCOF1* and *SALL1*. This highlights the need to identify other genes and genotype interactions that contribute to the etiology of craniofacial disorders, including microtia/OAVS [33]. Future research is also needed to assess the involvement of the RA pathway in the genetic etiology of microtia/OAVS.

## 5. Conclusions

Although gene–gene interactions are known to play an important role in the etiology of many complex diseases, no previous study has addressed gene interactions in patients with microtia/OAVS. Our finding of a gene interaction between *TCOF1* and *SALL1* in a group of Mexican patients with this entity supports the complex nature of ear embryogenesis and the development of microtia/OAVS. Further research is warranted, such as the inclusion of more candidate loci, which should lead to the identification of new gene–gene interactions underlying microtia/OAVS.

## Figures and Tables

**Table 1:** Evidence supporting the selection of the studied candidate microtia/OAVS genes.

Gene	Murine Knockout Models for Orthologous Genes with Microtia [5]	PV Have Been Identified in Familial Cases (AD or AR Inheritance) [17,18,19,20,21,37]	PV at These Loci Are Causative of Monogenic Syndromes That Present Microtia [4,5]	Expression during Ear Embryogenesis [38,39,40]	Participation in Retinoic Acid Pathway [23]
*EYA1*	+	NR	+	+	NR
*HOXA2*	+	+	+	+	+
*SALL1*	+	+	+	+	NR
*TBX1*	+	NR	+	+	NR
*TCOF1*	+	NR	+	+	NR

Abbreviations: + reported, PV pathogenic variants, AD autosomal dominant, AR autosomal recessive, NR not reported.

**Table 2:** Comparison of allelic frequencies (AFs) for the 17 non-synonymous gene variants of *TCOF1*, *SALL1*, *TBX1*, and *EYA1* identified in our population versus those reported in the reference group (Mexicans from LA, 1000 Genomes Project Database).

ACMG/AMP Classification: [Criteria] *					Cases in Our Study				Reference Group				
	Cases (n=)	cDNA	Protein	Reference SNP	HoRA	Hetero	HoMA	AF	HoRA	Hetero	HoMA	AF	*p*-Value
*TCOF1* NM_000356.3													
LB: [ BS1, BS2, BP1, BP4, BP6]	1	c.503C>T	p.(Thr168Met)	rs181203524	48	1	0	0.01	63	0	0	0	0.99
B: [BA1, BP1, BP4]	4	c.1762G>C	p.(Ala588Pro)	rs2071240	45	4	0	0.96	65	1	1	0.98	0.47
B: [BA1, BP1, BP4, BP6]	6	c.2429T>C	p.(Val810Ala)	rs7713638	43	6	0	0.94	57	9	1	0.92	0.55
B: [BA1, BP1, BP4, BP6]	4	c.3296C>G	p.(Pro1099Arg)	rs1136103	45	3	1	0.95	51	16	0	0.88	0.07
B: [BA1, BP1, BP4, BP6]	21	c.3938C>T	p.(Ala1313Val)	rs15251	28	20	1	0.78	38	22	7	0.73	0.45
B: [BP6, BS1, BS2, BP1, BP4]	1	c.4061G>C	p.(Gly1354Ala)	rs45491898	48	1	0	0.99	63	0	0	1	0.25
*SALL1* NM_002968.2													
B: [BS1, BS2, PP3]	1	c.400_411dup	p.(Lys134_Ser137dup)	rs750817837	48	1	0	0.01	64	0	0	0	0.99
B: [BA1, BP1, BP4, BP6]	4	c.475A>G	p.(Ser159Gly)	rs13336129	45	4	0	0.96	56	9	2	0.9	0.13
B: [BS1, BS2, PP3]	5	c.475_477del	p.(Ser159del)	rs113614842	44	5	0	0.95	65	0	0	1	**0.008**
LB: [BS1, BS2, BP1, BP4]	1	c.2804C>T	p.(Thr935Met)	rs755926434	48	1	0	0.01	63	0	0	0	0.99
B: [BS1, BS2, BP1, BP6, PP3]	1	c.3794G>A	p.Gly1265Glu)	rs149302006	48	1	0	0.01	63	1	0	0.007	0.84
B: [BA1]	49	c.3823G>A	p.(Val1275Ile)	rs4614723	0	0	49	0	0	0	67	0	NR
B: [BS1, BS2, BP1, BP4, BP6]	1	c.3872A>G	p.(Asn1291Ser)	rs74499562	48	1	0	0.01	62	2	0	0.015	0.72
*TBX1* NM_080647.1													
LB: [BP1, BP4, PP3]	1	c.68C>T	p.(Ala23Val)	rs1415687525	48	1	0	0.01	64	0	0	0	0.99
B: [BA1, BP1, BP4, BP6]	31	c.1189A>C	p.(Asn397His)	rs72646967	18	22	9	0.59	29	28	10	0.64	0.45
LB: [PM2, BS2, BP1]	1	c.1397C>T	p.(Ala466Val)	rs753613632	48	1	0	0.01	64	0	0	0	0.99
*EYA1* NM_000503.5													
LB: [BS2, BP6, PP2]	1	c.107C>T	p.(Thr36Ile)	rs727503048	48	1	0	0.01	64	0	0	0	0.99

Numbers in bold indicate *p*-value < 0.05. * Classified according to ACMG/AMP criteria [43]. Bracketed data indicates all criteria met by the variant. Abbreviations: AF: allelic frequency, HoRA: homozygous for the reference allele, Hetero: heterozygous, HoMA: homozygous for the minor allele, (n=): number of cases, NR: not previously reported.

**Table 3:** Detailed information on the synonymous variants observed in our population.

				Cases in Our Study				Reference Group				
Cases (n=)	cDNA	Protein	Reference SNP	HoRA	Hetero	HoMA	AF	HoRA	Hetero	HoMA	AF	*p*-Value
*TCOF1* NM_001135243.1												
1	c.630A>G	p.(Thr210=)	rs765654624	48	1	0	0.99	NR	NR	NR	NR	NR
5	c.1578C>T	p.(Pro526=)	rs2071238	44	5	0	0.95	54	9	1	0.91	0.32
1	c.1761G>T	p.(Gly587=)	rs7701163	48	1	0	0.99	56	8	0	0.94	**0.04**
5	c.1842A>G	p.(Ser614=)	rs2071239	44	5	0	0.95	54	9	1	0.91	0.32
*SALL1* NM_002968.2												
2	c.390G>A	p.(Pro130=)	rs75156807	47	2	0	0.98	62	2	0	0.98	0.78
1	c.570A>G	p.(Val190=)	rs1317946303	48	1	0	0.99	NR	NR	NR	NR	NR
1	c.1674G>A	p.(Pro558=)	rs747355231	48	1	0	0.99	NR	NR	NR	NR	NR
3	c.2178G>A	p.(Arg726=)	rs144019351	46	3	0	0.97	62	2	0	0.98	0.44
2	c.2343G>A	p.(Leu781=)	rs60270998	47	2	0	0.98	64	0	0	1	0.1
41	c.2574C>T	p.(Leu858=)	rs1965024	8	18	23	0.35	7	33	24	0.37	0.75
7	c.3456C>T	p.(His1152=)	rs11645288	42	5	2	0.91	50	12	2	0.88	0.47
*TBX1* NM_080647.1												
1	c.75G>T	p.(Gly25=)	rs72646952	48	1	0	0.99	62	2	0	0.98	0.72
1	c.135G>A	p.(Pro45=)	NR	48	1	0	0.99	NR	NR	NR	NR	NR
2	c.297G>A	p.(Ala99=)	rs72646953	47	2	0	0.98	62	2	0	0.98	0.78
31	c.420T>C	p.(Phe140=)	rs41298814	18	22	9	0.59	29	27	8	0.66	0.27
26	c.664C>T	p.(Leu222=)	rs2301558	23	24	2	0.71	37	22	5	0.75	0.53
31	c.933A>G	p.(Ala311=)	rs41298840	18	22	9	0.59	29	27	8	0.66	0.27
4	c.1059A>G	p.(Ala353=)	rs13054377	45	4	0	0.96	59	4	1	0.95	0.83
*EYA1* NM_000503.5												
1	c.585A>T	p.(Ile195=)	rs780672889	48	1	0	0.99	NR	NR	NR	NR	NR
22	c.813A>G	p.(Thr271=)	rs1445398	27	19	3	0.74	34	28	2	0.75	0.92
33	c.1278C>T	p.(Gly426=)	rs4738118	16	24	9	0.57	31	26	7	0.69	0.078
40	c.1755T>C	p.(His585=)	rs10103397	9	28	12	0.47	15	35	14	0.51	0.54

Reference group: Mexicans from LA, 1000 Genomes Project Database. Numbers in bold indicate *p*-value < 0.05. Abbreviations: AF: allelic frequency, HoRA: homozygous for the reference allele, Hetero: heterozygous, HoMA: homozygous for the minor allele, (n=): number of cases, NR: not previously reported.

## Data Availability

The genotypic datasets generated in this study have been uploaded to the Leiden Open Variation Database (LOVD) version 3.0 (https://www.lovd.nl/3.0/home, accessed on 20 September 2022).
